# Luminescence Thermometry Based on the Upconversion Luminescence from the Stark Sublevels of BaY_2_F_8_:Yb^3+^, Tm^3+^ Phosphor

**DOI:** 10.1007/s10895-023-03295-z

**Published:** 2023-07-14

**Authors:** Ashwini K. Sharma, Govind B. Nair, S. J. Dhoble, Robin E. Kroon, J. J. Terblans, H. C. Swart

**Affiliations:** 1 Department of Physics, University of the Free State, PO Box 339, Bloemfontein, 9300 South Africa; 2 Department of Physics, University, Dr. Shyama Prasad Mukherjee University, Ranchi, 834008 India; 3 Department of Physics, RTM Nagpur University, Nagpur, 440033 India

**Keywords:** Upconversion, fluoride, phosphor, Tm^3+^, luminescence thermometry

## Abstract

Visible and near-infrared (NIR) upconversion luminescence (UCL) emissions originating from the BaY_2_F_8_: Yb^3+^, Tm^3+^ systems were investigated under a laser excitation at 980 nm. The BaY_2_F_8_:20 mol% Yb^3+^, x mol% Tm^3+^ and BaY_2_F_8_: y mol% Yb^3+^, 0.5 mol% Tm^3+^ phosphors showed prominent UCL at 800 and 810 nm. The optimized doping concentrations of Yb^3+^ and Tm^3+^ in the BaY_2_F_8_ host matrix were evaluated, their spectroscopic properties were determined, and studies on their temperature-dependent behaviour were carried out. The temperature-sensing properties were studied by generating the fluorescence intensity ratio (FIR) of the UCL peaks originating from the thermally-coupled energy levels of the Tm^3+^ ions. The Stark sublevels of ^1^G_4_ level of Tm^3+^ ions were utilized to estimate the temperature-sensing abilities of the phosphor.

**Graphical Abstract:**

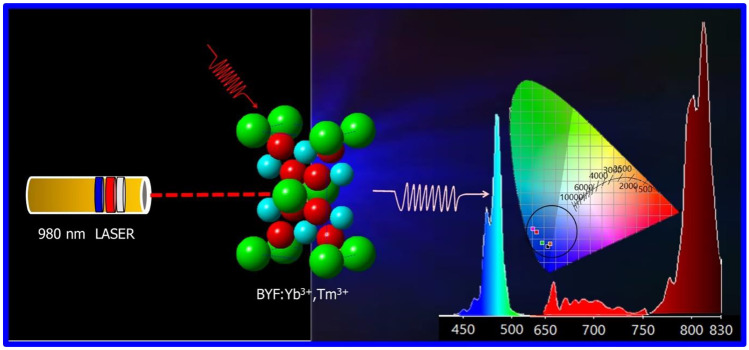

## Introduction

Lanthanide (Ln^3+^)-doped upconversion (UC) nanoparticles have been extensively studied for their versatile multifunctional characteristics especially for optical thermometers, lasers, displays, bioimaging, bio-sensing and therapy, solar spectral-convertors and other optical applications [1–4]. Among the UC materials, in order to achieve high UC efficiency, fluoride-based host matrices are preferred over other oxide hosts owing to their low phonon energies; e.g. BaYF_5_, NaYF_4_, and BaMgF_4_, etc. [5–7]. The selection of the host material also depends on the ease and suitability of the synthesis process. Several synthesis procedures have been adopted to prepare the UC materials, and the list includes the solid-state diffusion, hot-injection, microwave, hydrothermal, precipitation methods. While preparing fluorides, special attention is required to prevent the oxidation or formation of defects in the material. Such defects are not favorable for the UC processes, and it may lead to unwanted increase in the phonon energies that can increase the probability of non-radiative transitions in the lattice.

Fluorides are known to exhibit lower phonon energies that facilitate more efficient UC processes and eliminate the energy losses due to multi-phonon relaxations. Therefore, BaY_2_F_8_ (BYF) was selected as the host material in this work. The BaY_2_F_8_ host demonstrated excellent thermal stability and presented an intense green emission at 549 nm with the dopant pair of Yb^3+^-Ho^3+^ ion [8, 9]. Tm^3+^ ions are known to produce blue emissions along with much stronger NIR emissions. Due to the strong NIR UC emissions under 980 nm excitation, Tm^3+^-based UC phosphors can be used for thermometry of biological samples [10]. For improving the performance of the optical temperature sensors, UC phosphors with favorable crystal field environment around the Tm^3+^ ions and higher temperature sensitivity are required.

Luminescence thermometry is widely practiced as a reliable, durable and accurate form of remote thermometry [11]. The technique based on the fluorescence intensity ratio (FIR) is usually adopted to monitor the temperature variations. In this technique, the FIRs of the emission peaks originating from the thermally-coupled energy levels (TCELs) are determined at different temperatures. The FIRs of the TCELs follow Boltzmann distribution law [12, 13]. The energy spacing between the TCELs must lie between 200 and 2000 cm^− 1^ [14, 15]. Stark sublevels of Ln^3+^ ions also behave similar to TCELs; hence, the FIRs of Stark sublevels also contribute towards efficient temperature-sensing [4, 13]. In this study, the Stark sublevels of Tm^3+^ ions were used to study the temperature-sensing properties of BYF:Yb^3+^,Tm^3+^ phosphor. The FIRs of the Stark sublevels of ^1^G_4_energy level of Tm^3+^ were considered for investigating the temperature-sensing characteristics of BYF:Yb^3+^, Tm^3+^ phosphor in the temperature range 303 to 673 K.

## Materials and Methods

### UC Phosphor Synthesis

The synthesis procedure previously employed for the synthesis of BYF:Yb^3+^,Ho^3+^ was repeated for the preparation of BYF:Yb^3+^,Tm^3+^ phosphor [8]. BYF:Yb^3+^, Tm^3+^ phosphors were synthesized by the co-precipitation method at room temperature. The starting materials utilized were high purity (> 99.99%) chemicals of Ba(NO_3_)_2_, Y(NO_3_)_3_.6H_2_O, Tm(NO_3_)_3_, Yb(NO_3_)_3_, and NH_4_F that were procured from Sigma Aldrich. Stoichiometric amounts of the metal nitrates were dissolved in double distilled water and vigorously stirred on a magnetic stirrer. Few drops of 1-octadecene and oleic acid were added to this solution to stabilize the crystals in the solution. The phosphors were precipitated by adding the NH_4_F solution together with vigorous stirring. The precipitates were separated from the solution, and then, washed sequentially with water and ethanol. They were then dried in hot air oven for 12 h at 80 ^o^C. the dried precipitates were annealed in a reducing Ar/H_2_ (5% H_2_, 95% Ar gas mixture) atmosphere for 4 h at 650 ^o^C to obtain the final yield.

### Measurements and Characterizations

The X-ray powder diffraction (XRPD) patterns of the BYF:Yb^3+^,Tm^3+^ phosphors were recorded using a Bruker D8 Advance X-ray diffractometer in the 2θ ranging from 10 to 70^o^ with X-ray wavelength (λ) of 1.54 Å. The scanning electron microscope (SEM) images were obtained on a JSM-7800 F JEOL field emission scanning electron microscope (FE-SEM). The UCL spectra and the UC decay curves were recorded on a Edinburgh FLS 980 spectrofluorometer. The Commission Internationale de L’Eclairage (CIE) color coordinates were plotted using the Osram-Sylvania color calculator software. The temperature dependent UCL spectra were recorded using Horiba Jobin Yvon iHR320 Fully Automated Imaging Spectrometer. A 980 nm continuous-wave (CW) laser diode was used for pumping the phosphors while recording the UCL spectra, whereas a pulsed 980 nm laser was used for recording the UC decay curves.

## Results and Discussion

### Phase Formation and Morphology

**Fig. 1:**
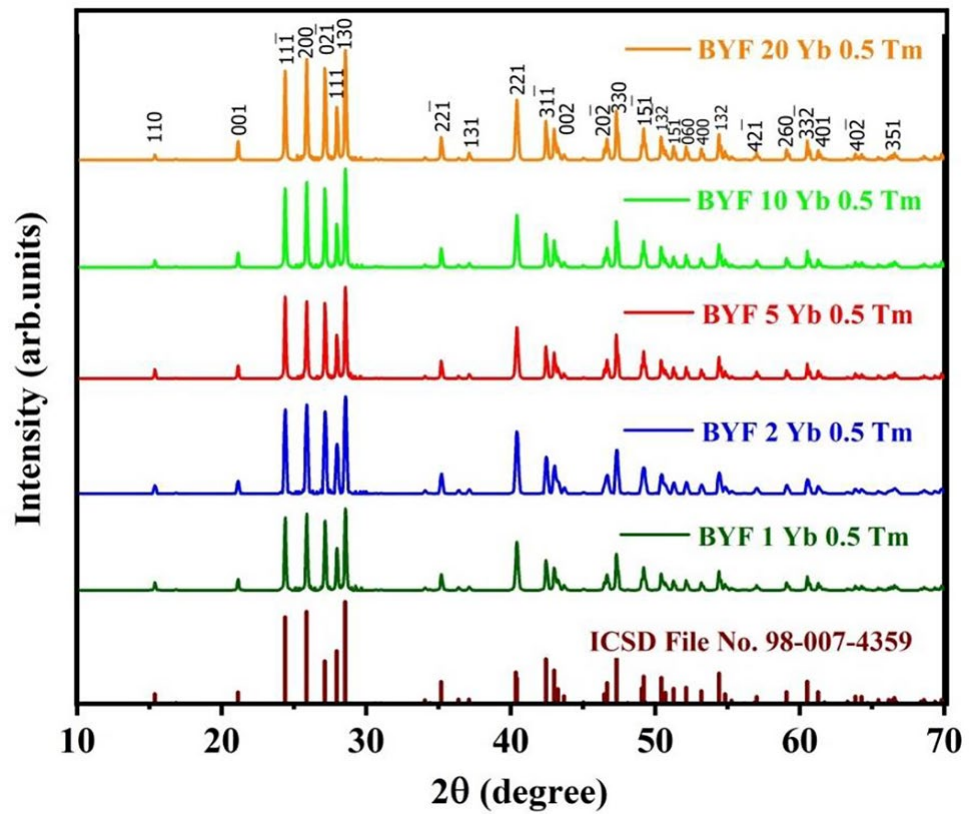
XRPD patterns of the BYF:y mol% Yb^3+^, 0.5 mol% Tm^3+^ (where, *y* = 1, 2, 5, 10 and 20) phosphor along with the standard pattern

Figure 1 shows the XRPD patterns of the powder samples of the BYF:*y* mol% Yb^3+^, 0.5 mol% Tm^3+^ (where, *y* = 1, 2, 5, 10 and 20) phosphor. The patterns confirmed the formation of the monoclinic phase for all the prepared samples, and they were found to be consistent with the ICSD file no. 98-007-4359 having space group *C 1 2/m 1*. None of these patterns indicated the presence of any impurity phases due to doping of the lanthanide ions, and thus, it was confirmed that all the samples were produced in a single-phase.

**Fig. 2:**
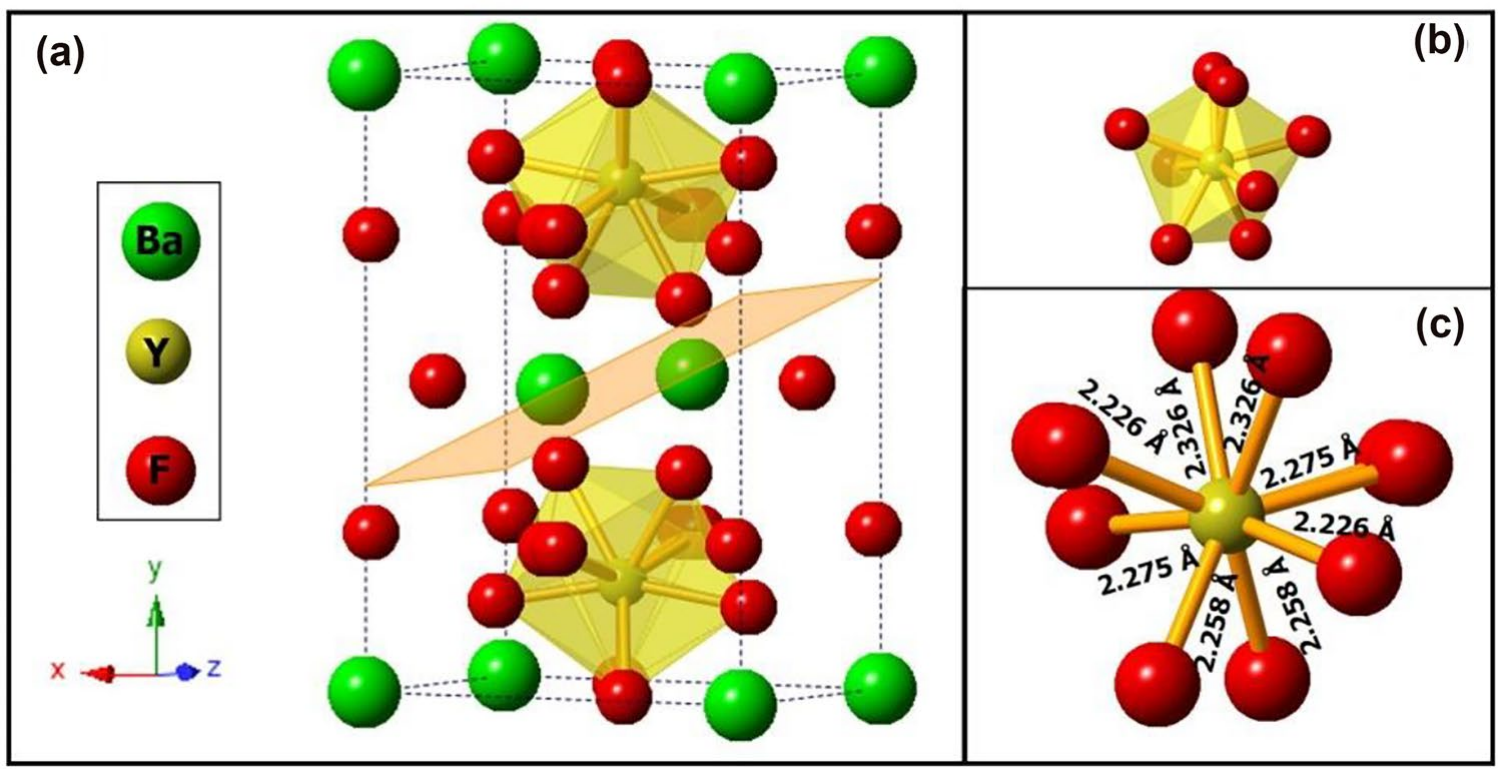
Monoclinic phase of the BYF host lattice constructed using the VESTA software

**Fig. 3:**
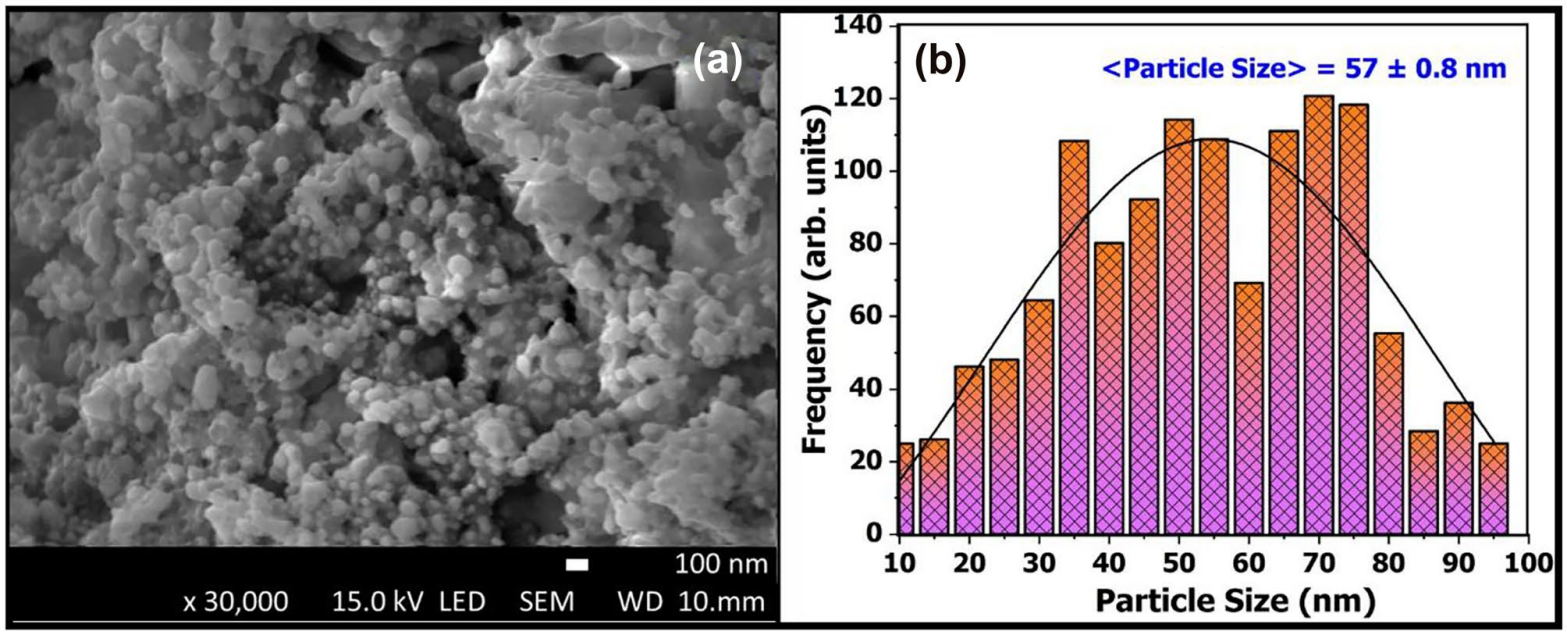
**a** SEM micrograph of the optimized Yb^3+^-Tm^3+^ codoped BYF phosphor. **b** Particle size distribution of the optimized (BYF 0.5Tm 5Yb) phosphor

The monoclinic crystal system of the BYF host lattice was modelled using the VESTA software, as shown in Fig. 2 [16]. The Ba^2+^ ions occupied the positions in the corner and the base-center along the z-axis of the structure. The six corner positions of the hexagonal plane appear to be occupied by the Y^3+^ ions, when perceived along the x-axis. Yb^3+^ and Tm^3+^ ions have also occupied some of the Y^3+^-sites due to their compatible ionic radii (Y^3+^ = 1.019 Å, Yb^3+^ = 0.985 Å, Tm^3+^ = 0.994 Å). Each of the Y^3+^-site is surrounded by eight fluoride ions. The detailed crystallographic parameters for the BYF host material is published in our previous research article [8]. The SEM micrograph of the optimized Yb^3+^-Tm^3+^ codoped BYF phosphor is shown in Fig. 3a. The surface morphology of the optimized UC phosphor (BYF 0.5Tm 5Yb) showed a large number of spherical particles that were agglomerated. The particle-size was interpreted using the ImageJ software [17, 18]. The particles were found to have dimensions in the nanometer range and the average particle size of the phosphor has been analyzed to be ~ 57 nm, as represented in Fig. 3b.

### Upconversion Luminescence (UCL) Spectra

**Fig. 4:**
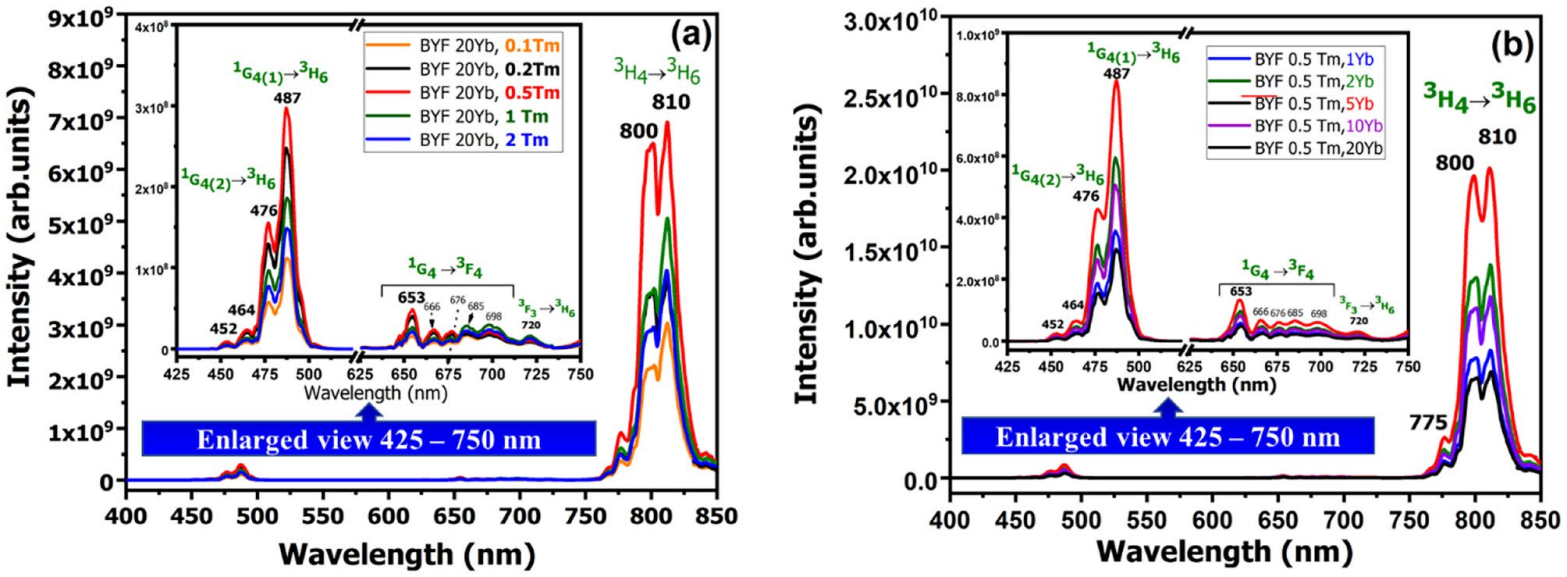
UCL spectra of: **a** BYF:20 mol% Yb^3+^, x mol% Tm^3+^ phosphor, and **b** BYF: y mol% Yb^3+^, 0.5 mol% Tm^3+^ phosphor from 400–850 nm and 425–750 nm region (enlarged view shown in inset)

**Fig. 5:**
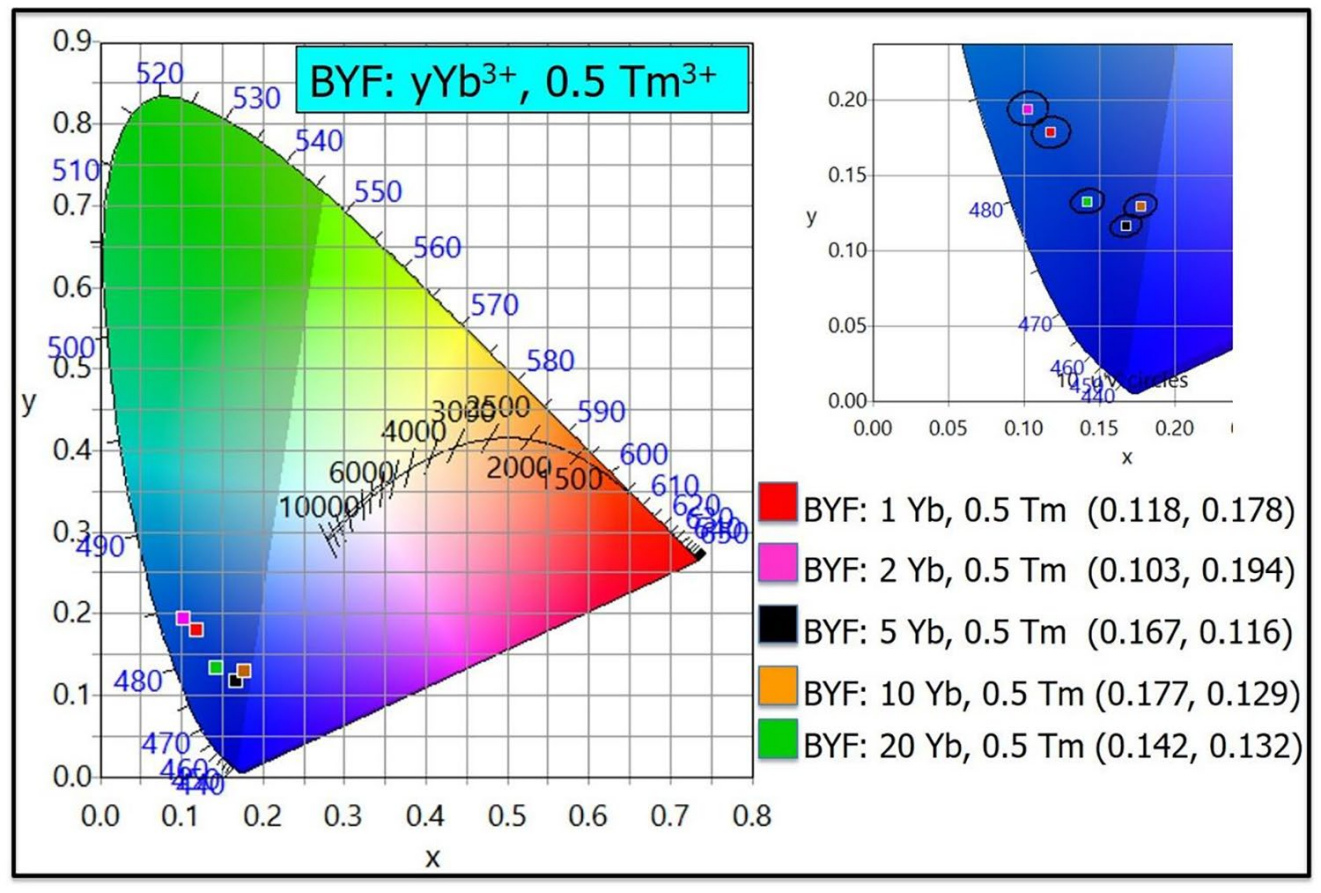
CIE chromaticity diagram for BYF: *y mol%* Yb^3+^, *0.5 mol%* Tm^3+^ (*y* = 1, 2, 5, 10 and 20) phosphors pumped by a 980 nm laser

The UCL spectra of two different series of BYF phosphors doped with Yb^3+^-Tm^3+^ pairs were measured by exciting the phosphors with a continuous-wave (CW) 980 nm laser diode operating at a fixed laser power of 1 W, as shown in Fig. 4a, b. Figure 4a presents the UCL emission spectra of BYF: 20 mol% Yb^3+^, *x* mol% Tm^3+^ (x = 0.1, 0.2, 0.5, 1 and 2), whereas Fig. 4b depicts the UCL spectra of BYF: *y* mol% Yb^3+^, 0.5 mol% Tm^3+^ (y = 1, 2, 5, 10 and 20). The optimized concentrations of the Yb^3+^ and Tm^3+^ ions in the BYF host were determined based on the luminescence intensities of the phosphors. In the first series, a high Yb^3+^-concentration was chosen as 20 mol% and the Tm^3+^-concentration was changed from 0.1 to 2 mol%. The optimum Tm^3+^-concentration was identified from the UCL spectra. The optimized Tm^3+^-concentration was kept as the fixed value and the Yb^3+^-concentration was varied from 1 to 20 mol% in the second series. The maximum UC emission was obtained for BYF: 20 mol% Yb^3+^, 0.5 mol% Tm^3+^ and BYF: 5 mol% Yb^3+^, 0.5 mol% Tm^3+^ in the first and the second series, respectively. The BYF: Yb^3+^,Tm^3+^ phosphor shows intense UCL emission bands for the ^1^G_4(2)_→ ^3^H_6_ (476 nm), ^1^G_4(1)_→ ^3^H_6_ (487 nm), ^3^H_4(2)_→ ^3^H_6_ (800 nm) and ^3^H_4(1)_→ ^3^H_6_ (810 nm) transitions. The other weak UCL peaks in the visible region were blue and red emissions attributed to the ^1^D_2_→ ^3^F_4_ (452 and 464 nm), ^1^G_4_→ ^3^F_4_ (653–698 nm), and ^3^F_4_ → ^3^F_6_ (720 nm) transitions of Tm^3+^. The intense NIR emission peaks at 800 and 810 nm are nearly 44 times stronger than the UCL intensity in the blue region. The CIE color coordinates for BYF: y mol% Yb^3+^, 0.5 mol% Tm^3+^ (*y* = 1, 2, 5, 10 and 20) phosphors were plotted in the CIE-1931 chromaticity diagram. Figure 5 shows the CIE diagram for the BYF: y mol% Yb^3+^, 0.5 mol% Tm^3+^ phosphors. Although the UCL spectra exhibited intense UCL in the NIR region, the integrated color emission was found to be in the blue region as the NIR wavelengths were not considered in the CIE diagram.

### Power Dependent UCPL

**Fig. 6:**
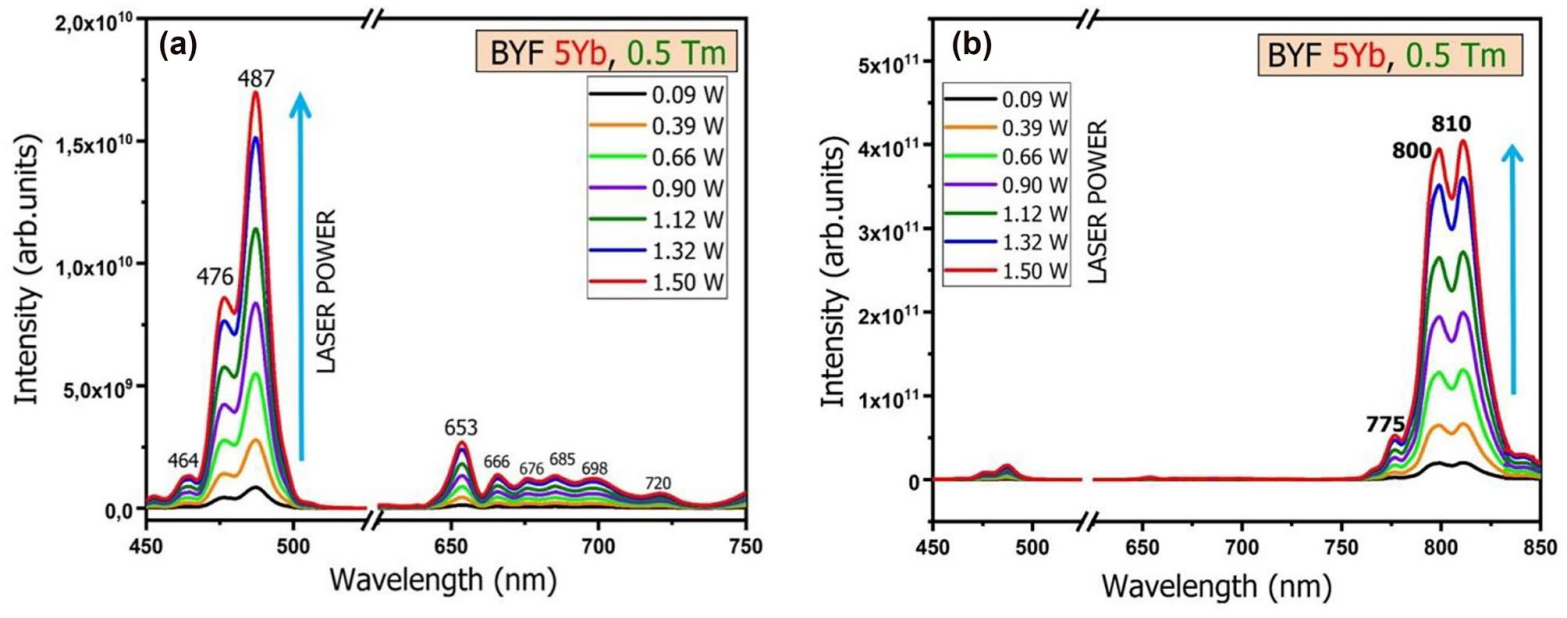
UCL spectra of BYF: 5 mol% Yb^3+^, 0.5 mol% Tm^3+^ phosphor excited by a 980 nm laser diode with different laser-powers ranging from 0.09 to 1.5 W at room temperature: **a** in the wavelength ranging from 450 to 750 nm, **b** in the complete wavelength range from 450 to 850 nm

**Fig. 7:**
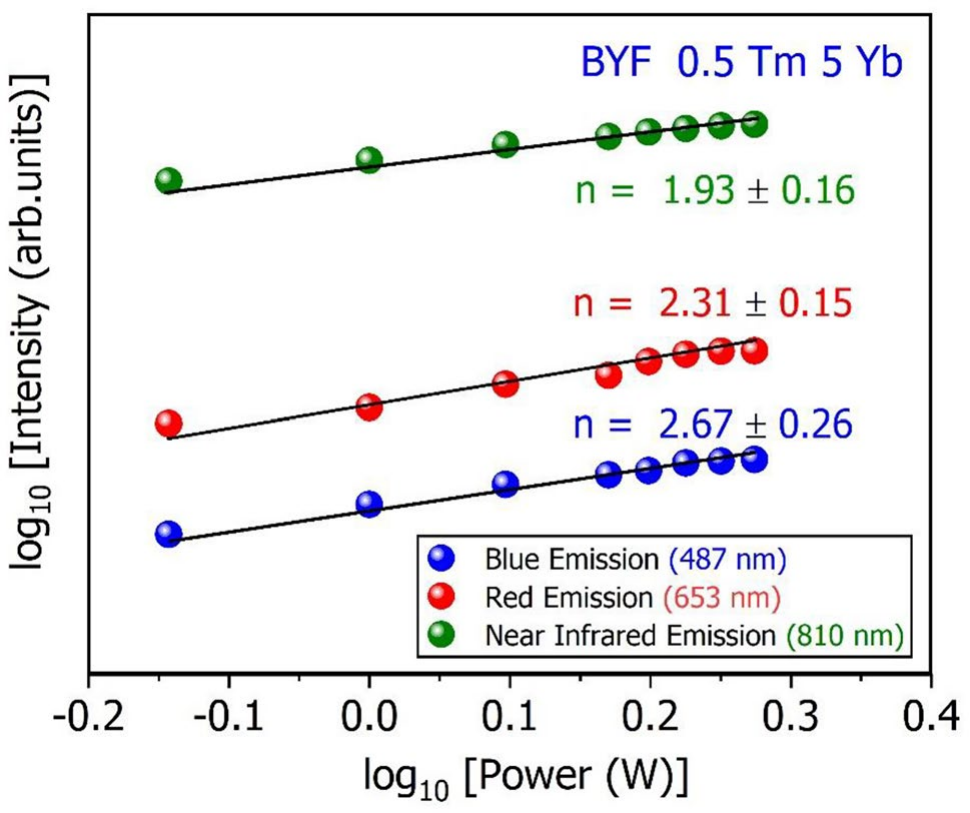
The logarithmic dependence of the UCL intensity as a function of the logarithmic value of the pump-power (laser energy) for BYF: 5 mol% Yb^3+^, 0.5 mol % Tm^3+^ phosphor at 487 nm, 653 and 810 nm emissions

The UCL spectra of the BYF: 5 mol% Yb^3+^, 0.5 mol% Tm^3+^ phosphor pumped by a 980 nm laser with different powers operating at room temperature is shown in Fig. 6. It can be seen that the UCL intensity increased monotonously with the increase in the pump-power. The UCL spectra do not show any degradation or additional peak with the variation in the pump- power. The number of pump photons necessary for the population of the upper emitting levels in the UC processes under unsaturated condition can be obtained by the relation [19]:

1 $$I_{UC}\;\infty\;P^n$$

where, *I*_*UC*_ is the integrated UCL intensity, *P* is the pump-power of the laser, and *n* represents the number of laser photons involved in the UC process. The value of *n* can be estimated from the slope of the linear-fit obtained from the logarithmic plot of the dependence of the UCL intensities [log(I_UC_)] on the laser pump-power [ log(P)]. The values of the slopes (*n*) measured for the blue emission (487 nm), red emission (653 nm) and NIR emissions (810 nm) were found to be 2.67, 2.31 and 1.93, respectively, as shown in Fig. 7. This indicates that a two-photon process was responsible for the NIR emissions, whereas a three-photon process was responsible for the blue and red emissions.

### Energy Transfer Mechanism

**Fig. 8:**
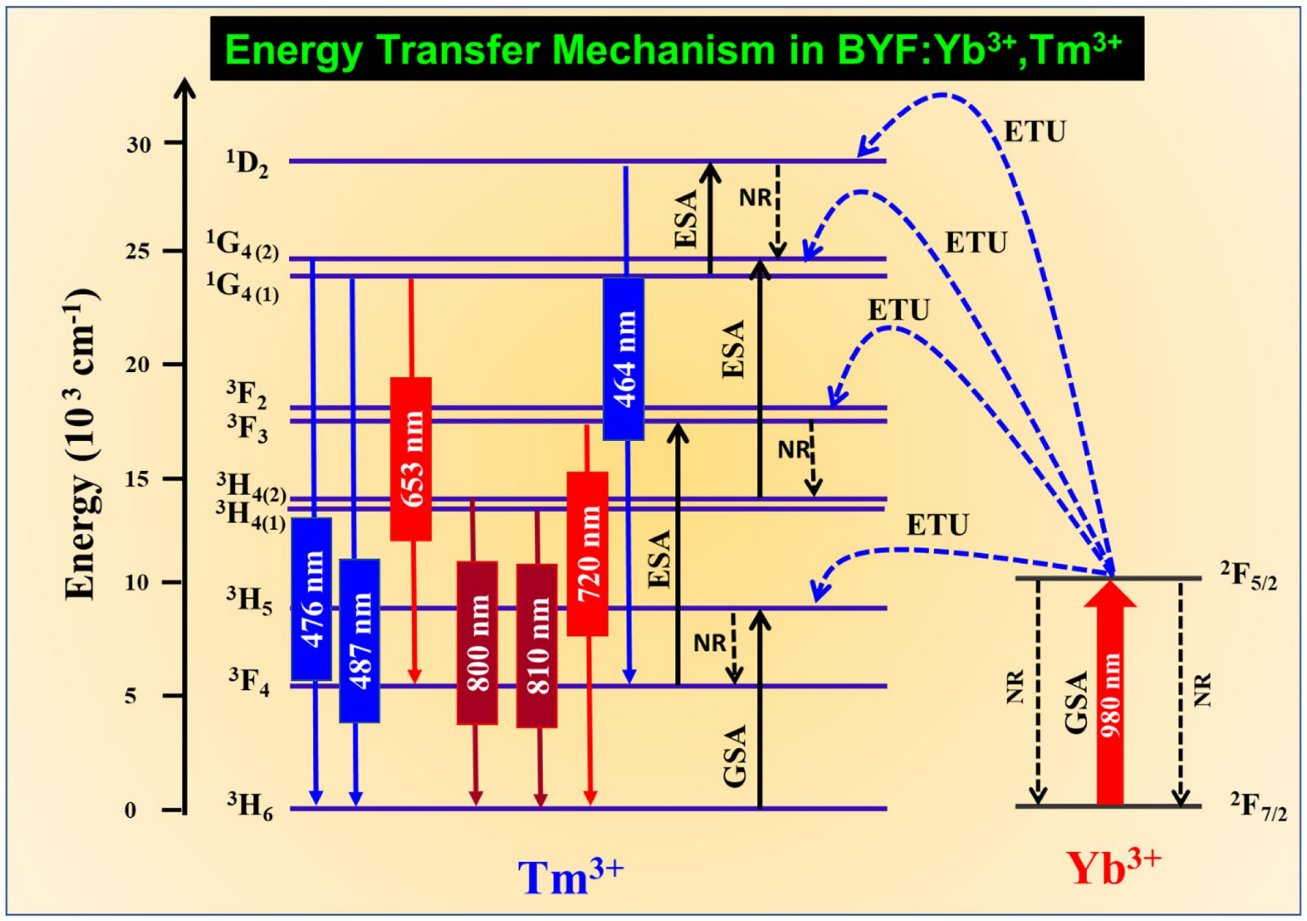
Schematic representation of the energy level diagram for BYF:Yb^3+^,Tm^3+^ system

The UCL can occur through several pathways in the Yb^3+^-Tm^3+^ system by different processes such as the ground state absorption (GSA), energy transfer upconversion (ETU), excited state absorption (ESA), and cross-relaxation (CR) [20]. Figure 8 shows the energy level scheme of processes that lead to the UCL of the BYF: Yb^3+^, Tm^3+^ phosphor. The most probable mechanism in the present UC phosphor is GSA and ETU because of the presence of two different luminescent ions. Here Yb^3+^ ions act as sensitizers that has much stronger absorption cross-section at 980 nm than the Tm^3+^ ions. Hence, Yb^3+^ can absorb the NIR radiations more efficiently and transfer them to the Tm^3+^ ions, which act as activator or luminescent centers in the host matrix. The Yb^3+^ ions in the ^2^F_7/2_ ground level get sufficient energy by absorbing the NIR (980 nm) photons and are then excited to the ^2^F_5/2_ level. The absorbed energy was non-resonantly transferred to the Tm^3+^ ions by the first step ETU process, which promoted the Tm^3+^ ions from the ground state to the ^3^H_5_ energy level. Another process by which the Tm^3+^ ions were promoted to this level was the GSA (^3^H_6_→ ^3^H_5_ transition). The ^3^H_5_ level non-radiatively relaxed to the ^3^F_4_ level. The ^3^F_4_ level was then promoted to the ^3^F_3_ level by the ESA process. Additionally, the ^3^F_3_ level was populated by the second step ETU process from the ^2^F_5/2_ level of the Yb^3+^ ions. The ^3^F_3_ level non-radiatively relaxed to the ^3^H_4_ level. The ^3^H_4_→^3^H_6_ transition generated radiative emissions in the NIR region. Again, the ^1^G_4_ state was populated by the ESA process through the ^3^H_4_→^1^G_4_ transition and the third ETU process between Yb^3+^ and Tm^3+^ ions. The radiative ^1^G_4_→^3^H_6_ and ^1^G_4_→^3^F_4_ transitions generated the blue and red emissions, respectively. The ESA process from the ^1^G_4_ level and the fourth ETU process from the ^2^F_5/2_ level of the Yb^3+^ ions resulted in the population of the ^1^D_2_ level of the Tm^3+^ ions. The radiative ^1^D_2_→^3^F_4_ transition also generated weak blue emissions at 452 and 464 nm. These radiative depopulation of the energy levels (^1^D_2_, ^1^G_4_, ^3^F_3_ and ^3^H_5_) in BYF: Yb^3+^, Tm^3+^ system resulted in the generation of UCL from Tm^3+^. The NIR peaks showed stronger emission intensity compared to blue peaks, which is a common feature observed in Yb^3+^/Tm^3+^ codoped fluoride-based phosphors [21, 22]. The NIR peaks originated from a low-lying energy level (^3^H_4_), whereas the blue emission peaks originated from a higher energy level (^1^G_4_). Tm^3+^ ions have a higher probability of populating the ^3^H_4_ levels through optical pumping as they are very close to the ground state. Also, it can be seen from the laser pump power dependence of the UCL intensity (Fig. 7) that only two pump photons were required to generate the NIR emission, whereas three pump photons were required to generate the blue emission. As a result, the emission intensity of the NIR peaks were stronger than the blue peaks.

### Decay Analysis

**Fig. 9:**
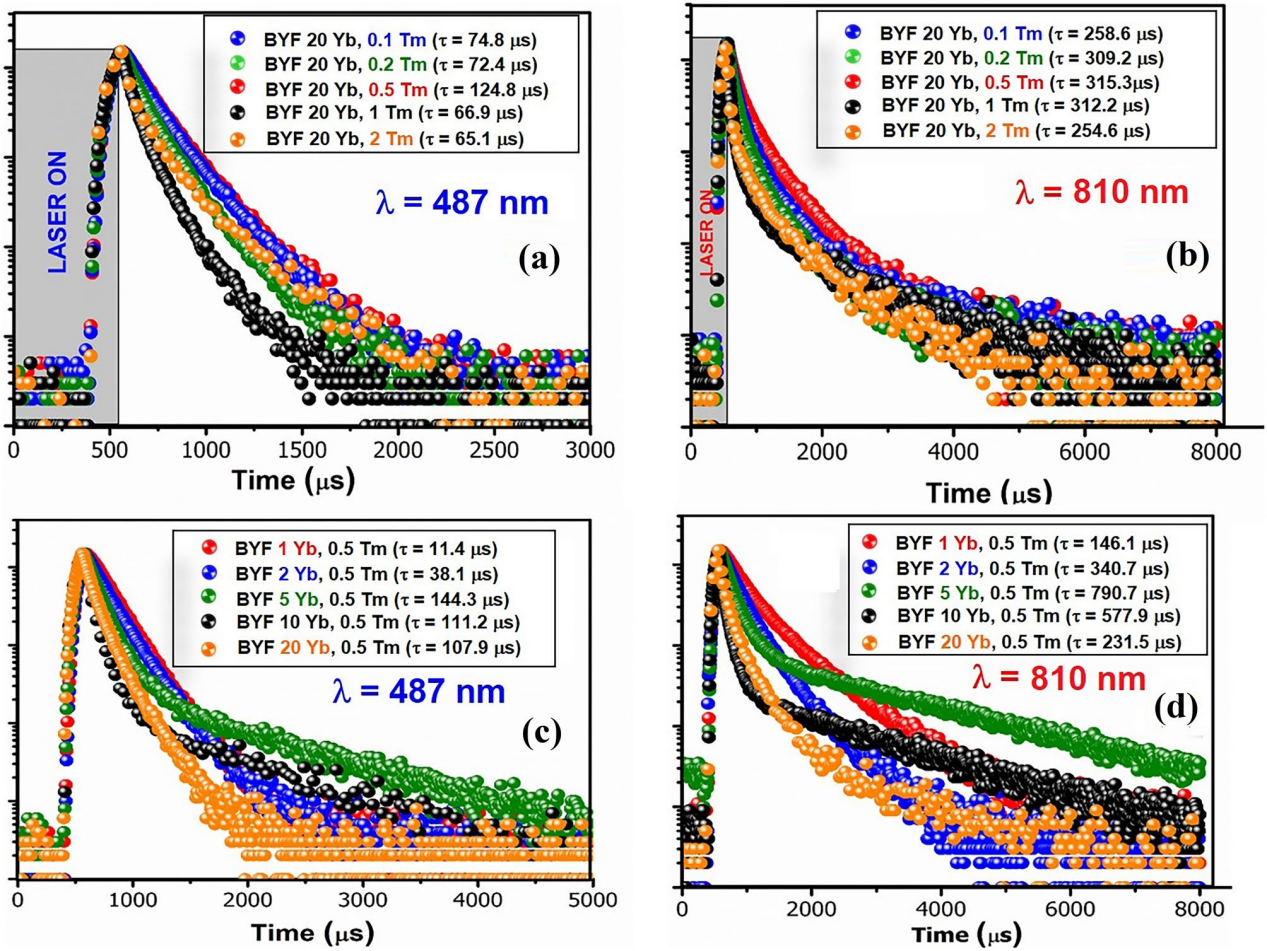
UC decay curves of BYF:Yb^3+^, Tm^3+^ phosphor excited by a 980 nm pulsed-laser for the Tm^3+^ emission at **a** and **c** 487 nm (^1^G_4_→ ^3^H_6_), and **b** and **d** 810 nm (^3^H_4_→ ^3^H_6_)

The UC decay curves of BYF: 20 mol% Yb^3+^, x mol% Tm^3+^ and BYF: y mol% Yb^3+^, 0.5 mol% Tm^3+^ phosphors were fitted with the double exponential function,

$$I\left(t\right)= {I}_{\infty }+{A}_{1}{e}^{\left(\frac{-t}{{\tau }_{1}}\right)}+{A}_{2}{e}^{\left(\frac{-t}{{\tau }_{2}}\right)}$$

where, A_1_ and A_2_ are the pre-exponential constants, τ_1_ and τ_2_ are the decay lifetimes, t is the time, I(t) is the UC intensity at time t, and ${\text{I}}_{{\infty }}$ is the background intensity, respectively. The above function was used to fit the decay curves and the average decay lifetime was obtained, as illustrated in Fig. 9. The decay curves recorded for the emission wavelengths 487 and 810 nm exhibited similar trend. There was a continuous increase in the decay lifetime with the Yb^3+^-concentration until y = 5. The decrease in the lifetime can be attributed to either the back transfer of energy from Tm^3+^→Yb^3+^ or due to the formation of Yb^3+^ pairs; both of these phenomena could have contributed to the luminescence quenching effect. The UCL quenching was also observed from the UCL spectra (Fig. 4), wherein the UCL intensity decreased after crossing the critical concentration of Yb^3+^ (or Tm^3+^).

### Thermal Stability Analysis

**Fig. 10:**
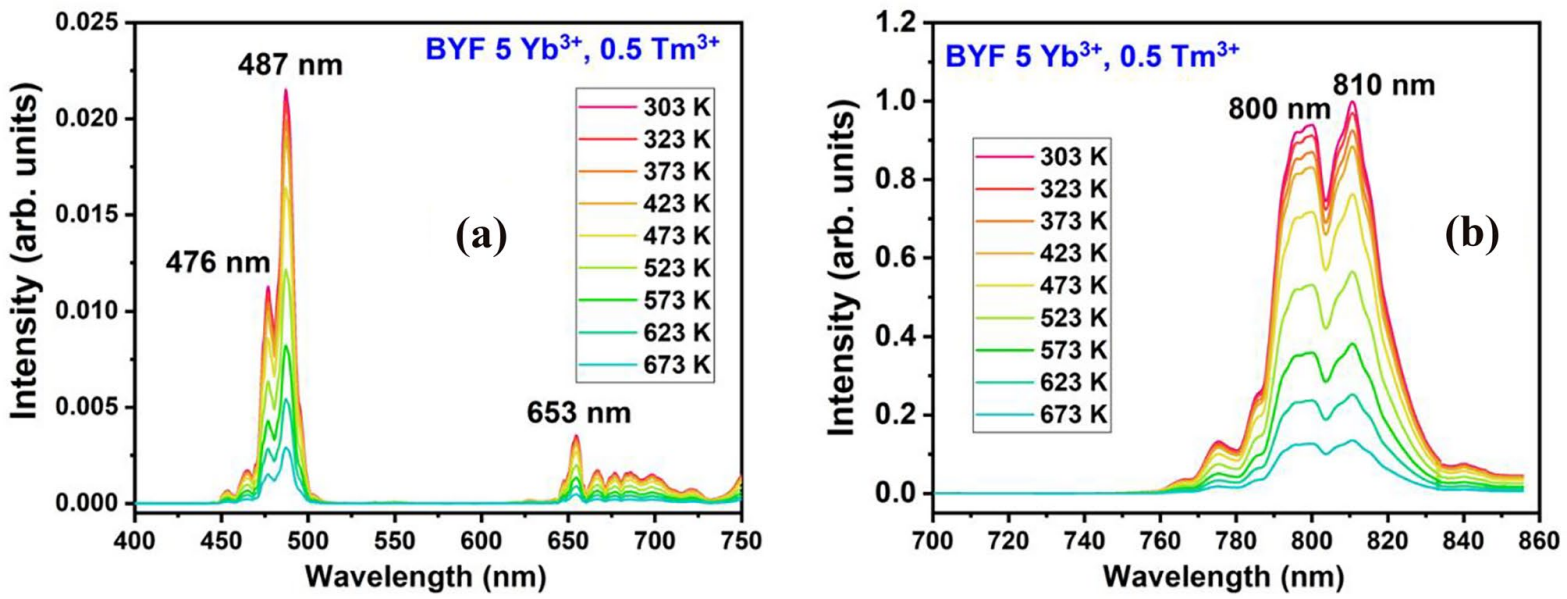
Temperature-dependent UCL spectra of BYF: 5 mol% Yb^3+^, 0.5 mol% Tm^3+^ phosphor: **a** Visible region, and **b** NIR region

**Fig. 11:**
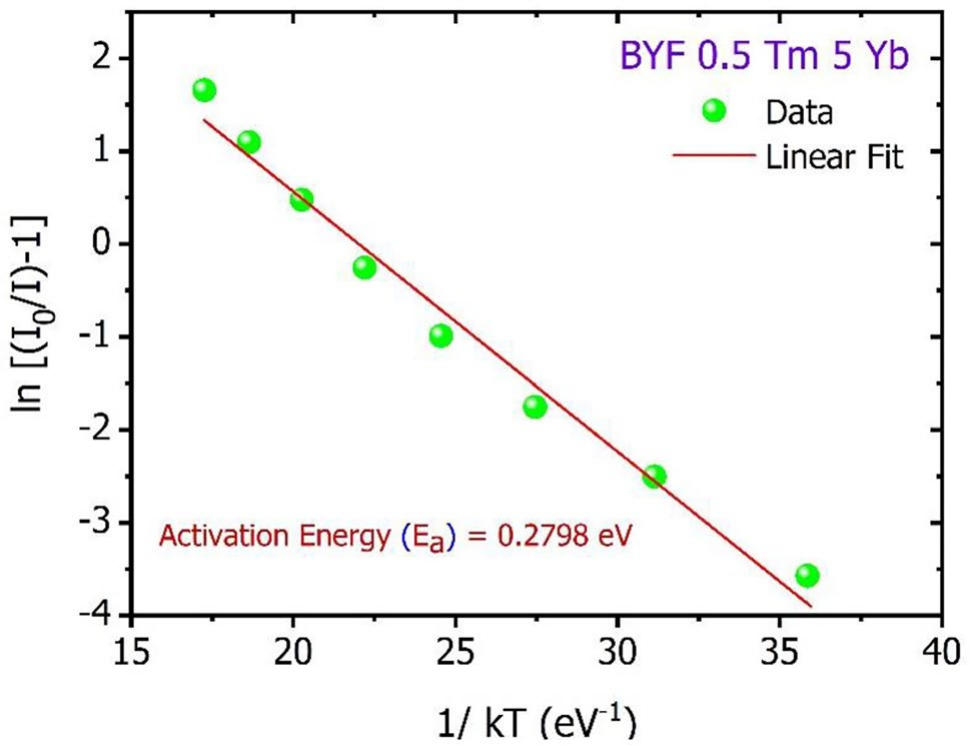
Activation energy (E_a_) plotted to determine the thermal quenching of BYF: 5 mol% Yb^3+^, 0.5 mol% Tm^3+^ phosphor

It is crucial to monitor the luminescence behaviour of a phosphor under stringent temperature conditions to test its durability before subjecting it to commercial applications. Here, the phosphor’s stability against the thermal variations was tested by recording the UCL spectra with the change in temperature. Figure 10 shows the temperature-dependent UCL spectra of BYF: 5 mol% Yb^3+^, 0.5 mol% Tm^3+^ phosphor excited by a 980 nm laser. The recordings were made for the temperatures ranging between 303 and 673 K. The increasing temperature has compelled the decrease in the UCL intensity due to the boosting of the population density of phonons giving rise to the electron-phonon energies in the BYF lattice. This led to the decrease in the number of radiative photons, and the prominence of the non-radiative energy transfers among the dopants [23, 24]. To elucidate the thermal quenching of the BYF: Yb, Tm phosphor, the temperature-dependent UCL emission intensity of the NIR peak was expressed through a simplified Arrhenius equation [25]:

$$\text{l}\text{n}\left(\frac{{I}_{0}}{I}\right)=\text{ln}\;A-\frac{{E}_{a}}{{k}_{B}T}$$

where, the meaning of the symbols are described elsewhere [8]. The activation energy plot for of BYF: 5 mol% Yb^3+^, 0.5 mol% Tm^3+^ phosphor is presented in Fig. 11. The plot was linearly fitted with the slope turning out to be 0.2798 eV. The value of the slope is equal to the thermal activation energy (E_a_) for the BYF: 5 mol% Yb^3+^, 0.5 mol% Tm^3+^ phosphor. The value of E_a_ was found to be 0.2798 eV for the BYF: 5 mol% Yb^3+^, 0.5 mol% Tm^3+^ phosphor. The temperature at which the UCL intensity reaches half its original value is considered as the thermal quenching temperature (T_50_). BYF:5 mol% Yb^3+^, 0.5 mol% Tm^3+^ phosphor retained 97%, 92%, 87%, 77% and 57% UCPL intensity at 323, 373, 423, 473, and 523 K, respectively. Thus, the T_50_ falls between 523 and 573 K.

### Temperature-sensing Behavior

**Fig. 12:**
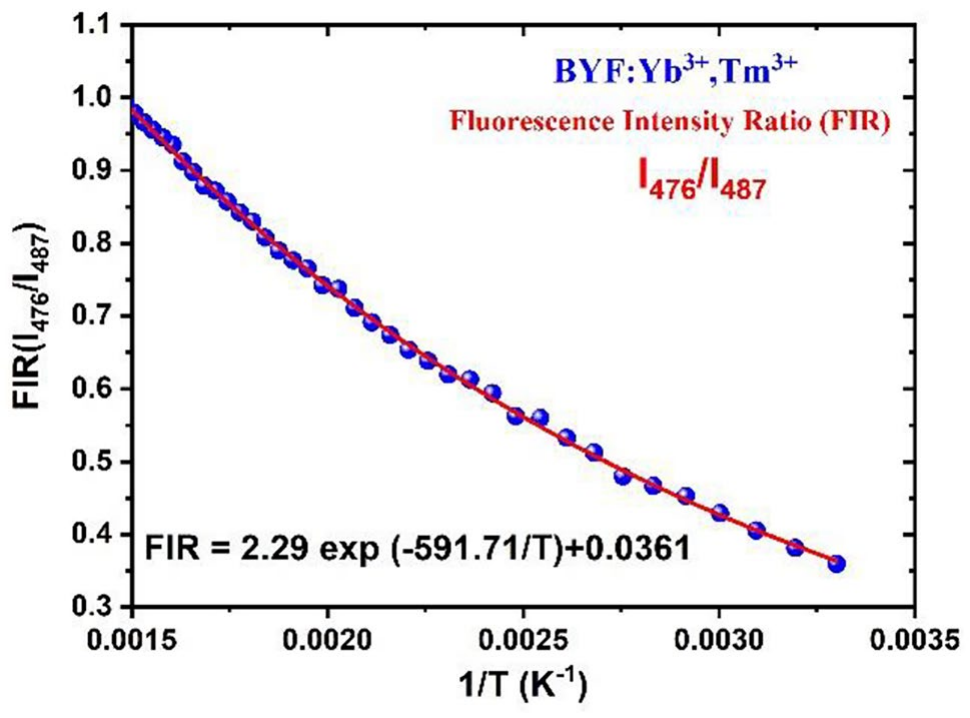
FIR versus 1/T plot of I_476_/I_487_ for BYF: 5 mol% Yb^3+^, 0.5 mol% Tm^3+^ phosphor

From the UCL, it was found that the emission peaks at 476 and 487 nm corresponded to the radiative transitions from the TCELs. The blue emissions at 476 and 487 nm originated from the Stark-split ^1^G_4_ levels. The Stark sublevels can also be considered as TCEls [4]. Using the Stark sublevels of the Tm^3+^ ion, the temperature-sensing behaviour of the BYF: 5 mol% Yb^3+^, 0.5 mol% Tm^3+^ phosphor was investigated. The fluorescence intensity ratios (FIR) were calculated between 476 and 487 nm. The temperature dependence of the FIR for the TCELs is expected to follow the trend:

$$FIR=A \;exp\;\left(\frac{-\varDelta E}{kT}\right)$$

where, the symbols have their usual meanings [4]. The logarithmic FIR plot for I_476_/I_487_ against T^− 1^ is shown in Fig. 12. The single-exponential decay function of the form:$y = {y}_{0}+A{e}^{\frac{-\varDelta E}{kT}}$, with the slope = $\frac{-\varDelta E}{k}$ was employed to fit the FIR versus T^−1^ plot. The slope of the FIR plot was found to be − 591.71, and the corresponding pre-exponential constant (*A*) was found to be 2.29. The relative (S_R_) and the absolute (S_A_) sensitivities of the BYF: 5 mol% Yb^3+^, 0.5 mol% Tm^3+^ phosphor was determined using the following equations:

$${ S}_{R}= \frac{1}{FIR}\frac{d\left(FIR\right)}{dT}=\left(\frac{-\varDelta E}{k{T}^{2}}\right)$$

$${S}_{A}= \frac{d\left(FIR\right)}{dT}=(FIR)*{S}_{R}$$

**Fig. 13:**
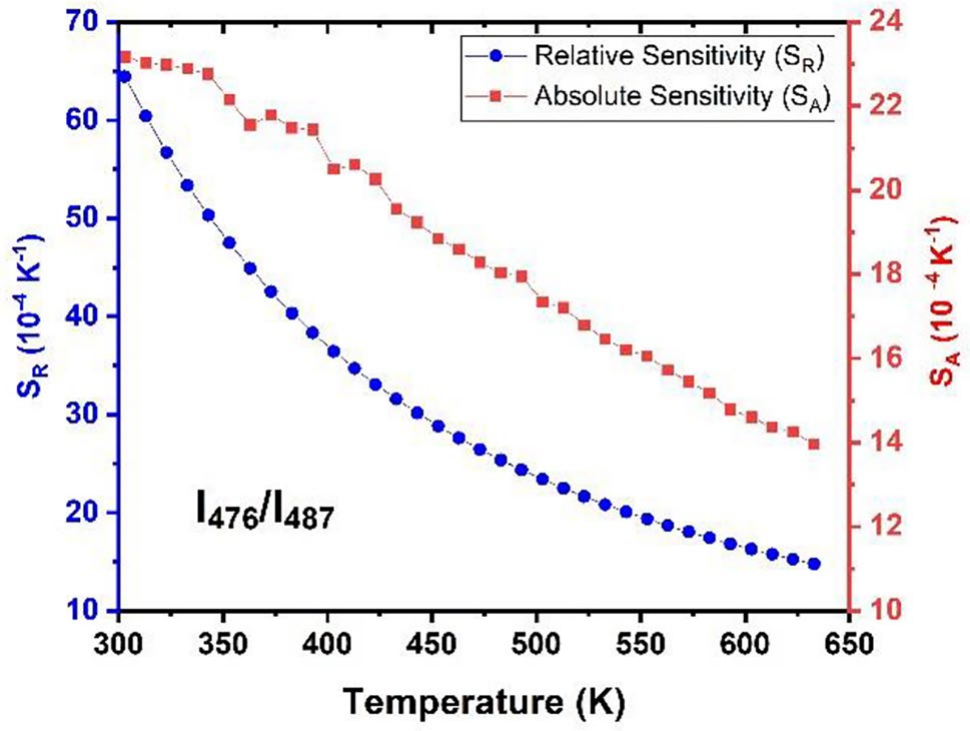
Variation of S_R_ and S_A_ for the BYF: 5 mol% Yb^3+^, 0.5 mol% Tm^3+^ phosphor as a function of temperature for the FIR plot of I_476_/I_487_

Figure 13 shows the temperature-dependent variation of S_R_ and S_A_ for the BYF: 5 mol% Yb^3+^, 0.5 mol% Tm^3+^ phosphor. The obtained values of S_R_ and S_A_ for certain specific temperatures are listed in Table 1. Both S_R_ and S_A_ exhibited their maximum values at the lowest temperature considered (303 K) and were found to decrease as the temperature increased.

**Table 1:** S_R_ and S_A_ at different temperatures for BYF: 5 mol% Yb^3+^, 0.5 mol% Tm^3+^phosphor

Sr. No.	Temperature (K)	I_476_ / I_487_	
		S_R_ (10^− 4^ K^− 1^)	S_A_ (10^− 4^ K^− 1^)
1	303	64.45	23.16
2	403	36.43	20.49
3	503	23.38	17.35
4	633	14.76	13.95

## Conclusion

The BaY_2_F_8_:Yb^3+^, Tm^3+^ phosphors were successfully obtained using the co-precipitation method and their UC process from NIR to NIR and blue photons have been demonstrated. On pumping the BaY_2_F_8_:Yb^3+^,Tm^3+^ phosphor with a 980 nm laser light, they showed characteristic UCL peaks viz. 476, 487, 653, 800 and 810 nm. The origin of the UC emissions were attributed to the ESA, CR and ETU between the Yb^3+^-Tm^3+^ ion pair through two and three photon processes. The optimum doping concentrations of the Yb^3+^ and Tm^3+^ were found to be 5 and 0.5 mol%, respectively. From the CIE diagram, the overall color emission of the phosphor was found in the blue region, despite the fact that the NIR emission peaks were multiple times more intense than the blue emission peaks. The phosphor was also found to be thermally stable with an activation energy equivalent to 0.2798 eV and the thermal quenching temperature beyond 523 K. The FIR of the UCL (I_476_/I_487_) was evaluated to check the temperature-sensing behaviour of the phosphor. At 303 K, the relative and absolute sensitivities of the phosphor were found to have their maximum values. The results suggest that the BaY_2_F_8_: 5 mol% Yb^3+^, 0.5 mol% Tm^3+^phosphor is a promising temperature sensor material that can be used in fire-safety alarms.

## Data Availability

No data sets were used in this work.
